# Preimplantation Genetic Testing for Aneuploidy Versus Morphological Selection in Women Aged 35–42: Results of a Pilot Randomized Controlled Trial

**DOI:** 10.3390/jcm14145166

**Published:** 2025-07-21

**Authors:** Yusuf Beebeejaun, Daniela Bakalova, Anastasia Mania, Timothy Copeland, Ippokratis Sarris, Kypros Nicolaides, Antonio Capalbo, Sesh K. Sunkara

**Affiliations:** 1 King’s Fertility, Fetal Medicine Research Institute, London SE5 8BB, UKsesh.sunkara@kcl.ac.uk (S.K.S.); 2 Department of Women’s and Children’s Health, Faculty of Life Sciences and Medicine, King’s College London, London SE1 7EH, UK; 3 School of Biosciences, University of Kent, Canterbury CT2 7NZ, UK; dany.bakalova@vitrolifegroup.com; 4 Department of Medicine, Division of Nephrology, University of California San Francisco, San Francisco, CA 94143, USA; 5 Harris Birthright Research Centre of Fetal Medicine, Fetal Medicine Research Institute, King’s College Hospital, London SE5 8BB, UK; 6 Unit of Molecular Genetics, Center for Advanced Studies and Technology (CAST), “G. d’Annunzio” University of Chieti-Pescara, 661003 Chieti, Italy

**Keywords:** preimplantation genetic testing, aneuploidy, embryo mosaicism, IVF

## Abstract

**Background/Objectives**: Embryo selection in IVF is traditionally based on morphology, yet many high-quality embryos fail to implant. Preimplantation genetic testing for aneuploidy (PGT-A) using next-generation sequencing (NGS) has been proposed to improve selection by identifying euploid embryos. However, its effectiveness in women of advanced maternal age remains unclear due to limited randomized data. This pilot trial assessed the feasibility of a full-scale RCT comparing PGT-A to morphology-based selection in women aged 35–42. **Methods**: This single-centre, two-arm parallel RCT (NCT05009745) enrolled women aged 35–42 undergoing IVF/ICSI with ≥3 good-quality day-3 embryos. Participants were randomized (1:1) to either embryo selection by morphology with fresh transfer or PGT-A with frozen transfer of a single euploid embryo. Allocation concealment was achieved via a secure web-based randomization platform; patients and clinicians were unblinded, but the biostatistician remained blinded. The primary outcome was feasibility of recruitment, randomization, and adherence. **Results**: Between June 2021 and January 2023, 138 women consented (recruitment rate: 55.8%, 95% CI: 49.7–62.0%) and 100 were randomized. Protocol adherence was 94%. Barriers to recruitment included preference for private PGT-A (19%) or fresh transfer (6%). Among biopsied embryos, 51.4% were euploid and 6.6% low-level mosaic. Intention-to-treat analysis showed no significant differences between PGT-A and control groups in clinical pregnancy rate (50% vs. 40%), live birth rate (50% vs. 38%), or miscarriage rate (12% vs. 8%). Cumulative live birth rate after up to three SETs was 72% vs. 52%, respectively (*p* > 0.05). No multiple pregnancies occurred. **Conclusions**: RCTs of PGT-A in older women are feasible. A multicentre design with broader inclusion criteria could improve recruitment and allow better assessment of clinical benefit.

## 1. Introduction

Embryo selection for in vitro fertilization (IVF) typically relies on morphological evaluation under a light microscope alone [1,2,3]. However, despite significant technological and scientific advances in assisted reproductive technology (ART), many women still fail to achieve clinical pregnancy or live birth, despite the transfer of a morphologically good embryo, thereby highlighting inherent limitations with morphological evaluation [4,5]. Choosing the correct embryo that will reduce the time to healthy live birth is of increasing clinical relevance in women with advancing reproductive age, where the incidence of aneuploidy can increase from 30% to 50% when a woman is 35 years old to 80% and over when aged 42 years or older [6,7]. To mitigate failed cycles, clinicians often transfer multiple embryos with good morphology, which increases the risk of multiple pregnancies and associated health risks to both mother and offspring [8].

Preimplantation genetic testing for aneuploidy (PGT-A) is often seen as a promising adjunct to conventional morphology-based selection. Supporters of PGT-A view this added embryo selection tool as a means to effectively identify and promote the transfer of single euploid embryos, with the highest potential for successful implantation and live birth, and reducing the risk of multiple gestations, while avoiding the transfer of aneuploid embryos which lead to failed pregnancy outcomes [9,10,11].

Nonetheless, the clinical efficacy of PGT-A remains contentious, particularly regarding improvements in live birth rates [12,13]. Initial studies utilizing fluorescence in situ hybridization (FISH) technology are considered obsolete due to limitations in chromosomal coverage and accuracy. Past PGT-A platforms also struggled with false positives/negatives and were unable to detect mosaicism—a common finding in IVF embryos [14]. Newer technologies, such as next-generation sequencing (NGS), provide a more comprehensive analysis of all 23 chromosomes [6,12,15].

Beyond these technical limitations, a fundamental biological concern is the phenomenon of ‘embryo correction’, where early embryos may have the capacity to self-correct chromosomal abnormalities as they develop through the clonal depletion of aneuploid cells and the propagation of karyotypically normal cell [16,17,18]. While advancements in technology may overcome some criticisms of PGT-A, the inherent variability introduced by embryo correction raises questions about the accuracy and ultimate utility of PGT-A in clinical practice.

Currently, there are limited trials assessing the clinical benefit of IVF with PGT-A via NGS vs. IVF with no PGT-A, and therefore the use of PGT-A prior to transferring embryos remains the epicentre of a perpetual controversy [19]. Challengers of PGT-A dismiss earlier trials due to concerns over the ‘intention-to-treat’ (ITT) criteria, focusing instead on the ESTEEM and STAR trials, which indicated that the use of PGT-A did not improve overall pregnancy outcomes in all women when analysed per embryo transfer or per ITT [12,20]. However, studies like the ESTEEM and STAR trials have their generalizability hindered by factors like the young average age of participants, which typically exhibit lower aneuploidy rates [12,20]. Supporters of PGT-A, on the other hand, state that, in the STAR trial, there was a significant increase in ongoing pregnancy rate (OPR) per embryo transfer with the use of PGT-A in the subgroup of women aged 35–40 years, where the rate of aneuploidy is higher and therefore screening is of higher clinical relevance. Critics, however, argue against the methodological rigor and conclusions of these studies, citing selection biases and design flaws [12,19,21].

Even the most recent multicentre study, by Yan et al., involving 1212 patients, which did not find that IVF with PGT-A yielded better outcomes than IVF without PGT-A [22], did not address these past criticisms. The study faced scrutiny over potential biases in methodology, average age of 29 years old, and choice of primary outcome measures, such as cumulative live birth rate, which may not fully capture the advantages of PGT-A with respect to clinical outcomes following first embryo transfer [23]. Additionally, the authors selected only three top-quality embryos for biopsy based on their morphology, rather than performing biopsies on all available embryos, which is standard clinical practice. Furthermore, the study did not consider the transfer of low-grade mosaic embryos, a procedure that can occur in real-world clinical settings [23,24]. As a result, the clinical effectiveness of PGT-A as a selection tool for women with a high expected aneuploidy rate, particularly those over the age of 35, is still to be established.

Our pilot study aimed to fill this gap by focusing on older patients, assessing the feasibility of conducting a multicentre RCT that explores PGT-A’s impact on clinical pregnancy and live birth rates in patients of advanced reproductive age, defined as aged 35–42. A key innovation of our study is its inclusion of mosaic embryos, which are frequently encountered but rarely studied in trials.

The proposed pilot study evaluated key operational components such as recruitment, randomization fidelity, adherence to protocols, and integration into a larger RCT framework.

We aimed to collect preliminary data to inform a definitive assessment of PGT-A coupled with NGS in enhancing IVF success, particularly within the high-aneuploidy-risk population of women aged 35–42 years. The goal of this pilot study and proposed future RCT design is to substantiate PGT-A’s role as a standard practice in this context, contributing to safer, more effective, and cost-efficient IVF treatments.

## 2. Materials and Methods

### 2.1. Trial Design

We conducted a prospective, allocation-concealed two-arm parallel unblinded RCT at King’s Fertility, part of King’s College Hospital in London, UK, from June 2021 to June 2023, as has been previously described [25]. The trial was approved by the East Midlands—Leicester South Research Ethics Committee (20/EM/0290) and registered at ClinicalTrials.gov of the United States National Institutes of Health (NCT05009745).

The study was reported in accordance with the extended guidelines of the Consolidated Standards of Reporting Trials (CONSORT) extended guideline for pilot and feasibility trials.

The trial was supported by funding from Ferring Ltd., with no control over the design and conduct of the study.

### 2.2. Study Population

Eligible participants were patients aged 35–42 years undergoing IVF ± ICSI treatment for treatment of infertility, with the aim of a fresh embryo transfer. Inclusion and exclusion criteria have been previously described [25].

Following eligibility confirmation, written informed consent and baseline data were obtained during face-to-face interviews.

Ovarian stimulation followed either a GnRH agonist downregulation or antagonist protocol, as per local practice. Final oocyte maturation was triggered once ≥2 follicles reached ≥18 mm using hCG, GnRH agonist, or both. Oocyte retrieval was performed 34–36 h later. All patients commenced vaginal progesterone for luteal support.

### 2.3. Randomization

Randomization occurred on day 3, as previously described [25], following egg collection if the patient had at least three transferrable embryos as assessed using the embryo scoring system of the Association of Clinical Embryologists (UK) and the Istanbul consensus [5,26,27]. 

### 2.4. Interventions

Control arm patients continued luteal support and underwent fresh blastocyst transfer on day 5 or elective freeze-all if clinically indicated. Surplus viable embryos were cryopreserved (day 5/6/7).

Experimental arm patients discontinued luteal support and underwent trophectoderm biopsy on all blastocysts (day 5/6/7). Samples were analysed using ReproSeq PGS kit (ThermoFisher Scientific, Waltham, MA, USA).

Embryos were cryopreserved post-biopsy; frozen embryo transfer occurred in the subsequent cycle based on PGT-A results.

Embryos were classified as low mosaic (30–50% aneuploid cells) or high mosaic (50–70% aneuploid cells). Only euploid or low mosaic embryos were transferred, with preference given to euploid embryos based on morphology.

Endometrial preparation for frozen transfers used natural, artificial, or ovulation-induction cycles, with luteal support per local practice as reported previously [28,29].

### 2.5. Outcomes

#### 2.5.1. Feasibility Outcomes

The primary feasibility outcome was the recruitment rate, defined as the number of participants randomized divided by the number assessed for eligibility. We aimed for a recruitment rate of at least 60%. Based on the methodological considerations for feasibility and the pilot study recommended by Shanyinde et al. [30], we established the following feasibility criteria:

1. A recruitment rate of 60%.
2. No more than 5% of recruited participants would withdraw consent.
3. At least 80% of eligible patients would be randomized.
4. An average of at least 5 subjects randomized per month.
5. At least 95% of the recruited participants would complete their final follow-up.

#### 2.5.2. Clinical Outcomes

Clinical outcomes included rates of clinical pregnancy rate (CPR), live birth (LBR), clinical miscarriage rate (CMR) per embryo transferred, and woman randomized after the initial embryo transfer.

A clinical pregnancy was defined as a pregnancy diagnosed by ultrasonographic visualization of one or more gestational sacs or definitive clinical signs of pregnancy [31]. Live birth was defined as a birth in which a fetus is delivered with signs of life after complete expulsion or extraction from its mother, beyond 20 completed weeks of gestational age [31]. A clinical miscarriage was defined as spontaneous loss of an intrauterine pregnancy prior to 20 completed weeks of gestational age [31]. Biochemical pregnancy loss, defined as an early pregnancy loss diagnosed only by the detection of beta hCG in serum or urine, were not included in our miscarriage calculations.

Cumulative live birth rates (cLBRs) following a subsequent maximum of 3 embryo transfer procedures were also recorded. The cumulative rates were calculated by dividing the number of women who had a live birth after transfers of a maximum of 3 blastocysts within 1 year after randomization by the total number of women who were assigned to the group.

### 2.6. Statistical Considerations

#### 2.6.1. Sample Size

For this pilot feasibility randomized controlled trial, a sample size of 100 women was chosen to allow estimation of recruitment rates with a margin of error of ±10.5% around the true rate, providing an 80% confidence interval [32].

While formal hypothesis testing is not the primary focus of this pilot study, an alpha value of 0.05 (5%) and a beta value corresponding to 80% power (β = 0.2) would be considered standard if preliminary hypothesis testing were conducted.

#### 2.6.2. Statistical Analysis

Continuous variables were summarized as means with interquartile ranges (IQRs) and standard deviations (SDs) and compared using the Wilcoxon rank-sum test. Categorical data were presented as frequencies and percentages, with comparisons via the Chi-squared test. Analyses excluded patients with missing covariates, and those lost to follow-up were considered not to have achieved live birth.

Outcomes were reported per intention-to-treat (ITT) and per-protocol (PP), with a secondary cumulative analysis across up to three embryo transfers. Clinical outcomes (CPR, LBR, CMR, cLBR) were presented as odds ratios (ORs) with 95% confidence intervals, both unadjusted and adjusted for age, BMI, nulliparity, and infertility cause. Statistical significance was set at *p* < 0.05 (two-sided); no corrections were made for multiple testing. Analyses were performed using STATA version 16 (STATA Corp., College Station, TX, USA).

## 3. Results

### 3.1. Participant Recruitment Flow

From June 2021 to January 2023, a total of 648 patients undergoing a cycle of IVF/ICSI were screened for eligibility. Of these, 247 patients were identified as eligible, yielding an eligibility rate of 38.2% (95% CI 34.5–41.9%). Among the eligible participants, 138 provided informed consent to participate in the study. The recruitment rate (138/237) was calculated at 55.8% (95% CI 49.7–62.0%), with an average of six patients being randomized each month.

A total of 38 patients did not meet our randomization criteria: 6 due to total fertilization failure and 32 due to having fewer than three high-quality embryos available by day three post-oocyte retrieval. Ultimately, 100 patients underwent randomization, with 50 women assigned to each study group. Adherence to the protocol was observed in 94% of participants.

In the intervention arm of the study (PGT-A), 46 patients received their allocated treatment, 2 patients did not have any euploid embryos available for transfer, 1 withdrew her consent, and another patient fell spontaneously pregnant awaiting a frozen embryo transfer.

In the control arm of the study, 48 patients adhered to the intended protocol assigned, 1 withdrew her consent from the study, and another one fell spontaneously pregnant awaiting a frozen embryo transfer, which was recommended due to her developing early ovarian hyperstimulation syndrome (OHSS).

**Figure 1:**
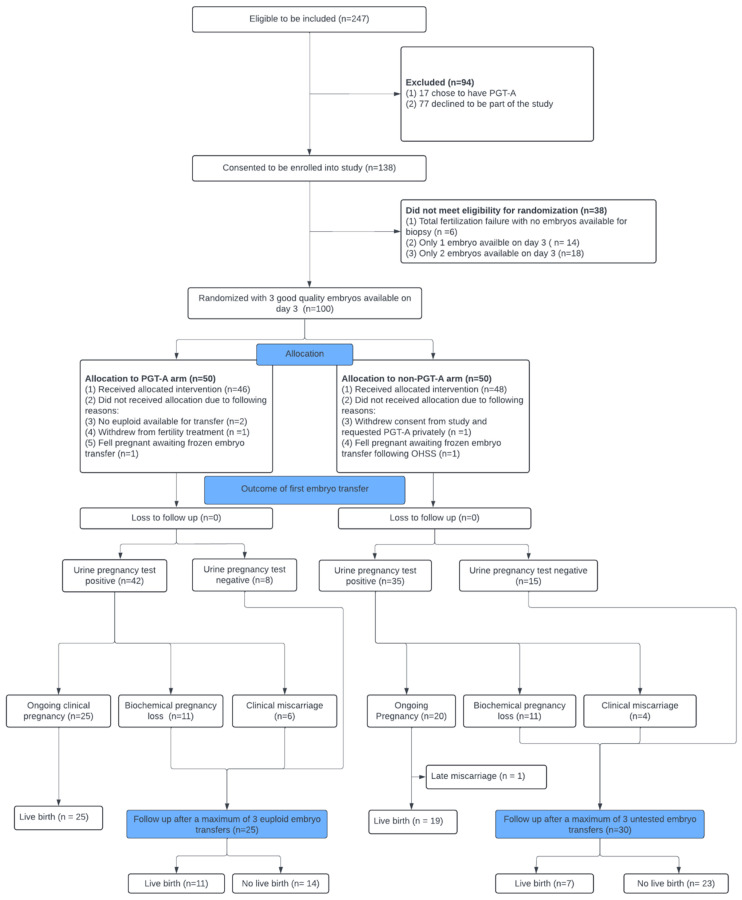
Patient flow diagram outlining recruitment, randomization, treatment allocation, and follow-up in the trial assessing the effectiveness of PGT-A on clinical outcomes.

Of those who had an embryo transfer, 100% completed the follow-up until their pregnancy test was performed (Figure 1).

### 3.2. Patient Characteristics

The baseline characteristics, including female age and ethnicity, male age and ethnicity, cause of infertility, anti-Müllerian hormone (AMH) levels, body mass index (BMI) of female patient, obstetrics history, average dose of gonadotrophins, numbers of days of ovarian stimulation, number of oocytes retrieved, number of mature oocytes, mode of insemination, and number of embryos available for transfer and biopsy in both groups are reported in Table 1.

There was no statistically significant difference in the baseline characteristics between either arm of the study.

### 3.3. Embryo Characteristics

A total of 571 blastocysts were morphologically available for transfer with 259 analysed by PGT-A with NGS. A total of 133 (51.4%) were euploid, 88 (34.0%) were aneuploid, 14 were classified as low-mosaic (5.41%), 3 as high-mosaic (1.15), 10 (3.86%) were reported as being chaotic, 7 (2.70%) had no DNA detected, and 4 (1.5%) were inconclusive.

Two women in the PGT-A group (4%) had only aneuploid embryos and therefore did not have an embryo transfer in the included cycle. Of the 50 women in each group, 2 (4%) in the PGT-A group and 2 (4%) in the conventional-IVF group withdrew from the trial or had a deviation from the protocol (Figure 1).

### 3.4. Clinical Outcomes as per Intention-to-Treat Analysis

The results of the intervention based on the intention-to-treat analysis of both groups are shown in Table 2.

#### 3.4.1. Clinical Pregnancy Rate (ITT Analysis)

The CPR was 50% (25/50) in the PGT-A arm and 40% (20/50) in the non-PGT-A arm (unadjusted OR 1.39, 95% CI: 0.64–3.03; *p*-value = 0.44; adjusted OR 1.31, 95% CI: 0.59–2.29; *p*-value = 0.52).

#### 3.4.2. Clinical Miscarriage Rate (ITT Analysis)

Clinical miscarriage rates of 12% (6/50 women) and 8% (4/50) were reported in the PGT-A arm and non-PGT-A arm, respectively (unadjusted OR 1.57, 95% CI: 0.41–5.94; *p*-value = 0.51; adjusted OR 1.20, 95% CI: 0.52–2.79; *p*-value = 0.83).

#### 3.4.3. Live Birth Rate (ITT Analysis)

LBRs of 50% (25/50 women) and 38% (19/50 women) were reported in the PGT-A arm and non-PGT-A arm, respectively (unadjusted OR 1.63, 95% CI: 0.74–3.62; *p*-value = 0.23; adjusted OR 1.47, 95% CI: 0.61–3.54; *p*-value = 0.39).

#### 3.4.4. Cumulative Live Birth After a Maximum of Three Embryo Transfers (ITT Analysis)

A total of 55 women underwent subsequent embryo transfers: 25 within the PGT-A arm and 30 within the non-PGT-A arm.

Cumulative LBRs of 72% (36/50 women) and 52% (26/50 women) were reported in the PGT-A arm and non-PGT-A arm, respectively (unadjusted OR 2.37, 95% CI: 1.04–5.44; *p*-value = 0.04; adjusted OR 2.26, 95% CI: 0.91–5.60; *p*-value = 0.08).

### 3.5. Clinical Outcomes as per Protocol Analysis

The results of the per-protocol analysis were consistent with those of the ITT analysis.

#### 3.5.1. Clinical Pregnancy Rate (PP Analysis)

The CPR was 50% (25/48) in the PGT-A arm and 40% (20/46) in the non-PGT-A arm (unadjusted OR 1.46, 95% CI: 0.65–3.28; *p*-value = 0.41; adjusted OR 1.38, 95% CI: 0.59–3.24; *p*-value = 0.46).

#### 3.5.2. Clinical Miscarriage Rate (PP Analysis)

Clinical miscarriage rates of 12.5% (6/48 women) and 8.7% (4/46) were reported in the PGT-A arm non-PGT-A arm, respectively (unadjusted OR 1.61, 95% CI: 0.42–6.12; *p*-value = 0.52; adjusted OR 1.92, 95% CI: 0.47–7.79; *p*-value = 0.36).

#### 3.5.3. Live Birth Rate (PP Analysis)

LBRs of 52.1% (25/48 women) and 41.3% (19/46 women) were reported in the PGT-A arm and non-PGT-A arm, respectively (unadjusted OR 1.59, 95% CI: 0.71–3.59; *p*-value = 0.26; adjusted OR 1.49, 95% CI: 0.61–3.66; *p*-value = 0.38).

#### 3.5.4. Cumulative Live Birth After a Maximum of Three Embryo Transfers (PP Analysis)

Cumulative LBRs of 75.0% (36/48 women) and 56.5% (26/46 women) were reported in the PGT-A arm and non-PGT-A arm, respectively (unadjusted OR 2.47, 95% CI: 1.04–5.87; *p*-value = 0.04; adjusted OR 2.50, 95% CI: 0.96–6.51; *p*-value = 0.06).

### 3.6. Patients Who Did Not Meet Randomization Criteria

Thirty-eight patients failed to meet our randomization criteria due to either fertilization failure resulting in no embryos being available (15.7%, 6/38), or having only one (7.89%%, 14/38) or two transferrable (42.1%, 16/38) good-quality embryos available for biopsy on day 3 post oocyte collection.

The baseline characteristics comparing patients who met our randomization criteria vs. patients who did not meet our randomization criteria are reported in Supplementary Table S1.

The average age of patients who failed to meet randomization was 39 years vs. an average of 37 within the pilot study (*p* value < 0.001); the average AMH value of patients who failed to meet randomization was 8.2 pmol/L (Median Q1: 5.3 pmol/L, Median Q3: 14.2 pmol/L) compared to an AMH of 17.2 (Median Q1: 10.2 pmol/L, Median Q3: 28.9 pmol/L) within the pilot study (*p* value < 0.001).

Patients who failed to meet randomization also had a higher dose of gonadotrophins prescribed (375 IU, Median Q1: 300 IU, Median Q3: 450 IU) compared to patients who met the randomization criteria (300 IU, Median Q1: 225IU, Median Q3: 375 IU) with lower number of oocytes retrieved (7.0 vs. 17), lower number of mature oocytes (3 vs. 10), and lower number of embryos available (1 vs. 5) (*p* value < 0.001).

Compared to all patients who were randomized, patients found ineligible for randomization had statistically significantly lower CPR 15.7% (6/38) vs. 45% (45/100) (unadjusted OR 6.97, 95% CI: 3.04–16.01; *p*-value < 0.001; adjusted OR 5.80, 95% CI: 2.07–16.25; *p*-value = 0.001) and LBR 7.89% (3/38) vs. 44.0% (44/100) (unadjusted OR 8.90, 95% CI: 2.56–30.92; *p*-value = 0.001; adjusted OR 5.90, 95% CI: 1.47–23.61; *p*-value = 0.01), respectively (Supplementary Table S2).

Compared to patients who were randomized and had a euploid embryo transfer, patients found ineligible for randomization had statistically significantly lower CPR 15.7% (6/38) vs. 50% (25/50) (unadjusted OR 6.00, 95% CI: 2.00–17.97; *p*-value < 0.001; adjusted OR 7.44, 95% CI: 1.74–31.85; *p*-value < 0.05) and LBR 7.89% (3/38) vs. 50% (25/50) (unadjusted OR 10.7, 95% CI: 2.89–39.41; *p*-value < 0.001; adjusted OR 12.6, 95% CI: 2.42–65.9; *p*-value < 0.05), respectively (Supplementary Table S2).

## 4. Discussion

### 4.1. Feasibility

This pilot study demonstrated the feasibility of conducting a two-arm randomized controlled trial (RCT) to evaluate the clinical benefits of PGT-A using NGS in patients aged 35 to 42 years. The trial achieved four of the six predefined feasibility criteria, including the recruitment of participants per month, a withdrawal rate of less than 5%, minimum compliance with interventions, and follow-up compliance.

The recruitment rate was 55.8% (138/247) below the targeted 80% over a 24-month period. This was largely attributable to the fact that many individuals opted to pursue PGT-A as part of their treatment after reviewing the patient information leaflet. Out of those who consented, 100 were randomized (72.4%, 95% CI: 65.0–79.9%). Future trials need to reflect on having strict inclusion criteria, as they may inadvertently select a subgroup of patients who possess a more favourable prognosis relative to the broader population typically encountered in clinical practice. Consequently, the outcomes observed in RCTs may not accurately reflect the challenges faced by the majority of IVF patients, particularly those with more compromised reproductive potential.

For future studies, broadening the inclusion criteria or designing complementary trials that capture a more representative patient cohort will be essential to ensure that the results are applicable to real-world practice and can inform routine clinical decision-making.

### 4.2. Clinical Outcomes

We observed a higher cumulative live birth rate (cLBR) in the PGT-A arm compared to the control group in our intention-to-treat analysis; however, this difference did not reach statistical significance. These findings should be interpreted cautiously, given the study’s limited sample size and its exploratory nature. It is also important to note that the PGT-A arm employed a dual selection strategy—first selecting for euploidy and then prioritizing embryos based on morphology—which may contribute to the observed trend. The absence of statistical significance likely reflects the sample size, baseline patient characteristics, and limitations inherent to aneuploidy screening, including the potential effects of embryo mosaicism and self-correction.

While this pilot study was not powered to evaluate clinical outcomes definitively, these exploratory findings highlight the complexity of embryo selection strategies and emphasize the need for larger, adequately powered trials to clarify the potential role of PGT-A in optimizing live birth outcomes.

It should also be noted that fresh embryo transfers were performed in the control group and frozen-thawed transfers in the PGT-A group. While some studies suggest differences in specific subgroups, large trials in women with normal ovarian response or advanced maternal age have shown comparable live birth rates between fresh and frozen cycles [33,34,35]. Given the moderate ovarian response in our cohort, this difference is unlikely to have significantly impacted our findings, although it remains an important consideration for future studies.

In our trial, clinical pregnancy was achieved after the first embryo transfer in 82% of women in the PGT-A group, compared to 70% in the control group. Although this showed a favorable trend, the difference was not statistically significant. While our pilot study focused on clinical pregnancy, live birth remains the most meaningful patient-centred outcome and should be prioritized as a primary endpoint in future RCTs.

### 4.3. Mosaicism and Embryo Selection Strategies

In our study, embryo selection was based on a hierarchical approach favoring the transfer of euploid embryos first, followed by low-mosaic embryos, then high-mosaic embryos, while aneuploid embryos were excluded from transfer. However, since our trial was designed, the latest studies suggest that low-mosaic embryos exhibit implantation and live birth rates comparable to euploid embryos [36], leading to a shift in clinical practice where low-mosaic embryos are increasingly regarded as functionally euploid [37]. Given this evolving perspective, future trials should consider refining embryo selection strategies by categorizing embryos based solely on euploid vs. aneuploid status, rather than incorporating mosaicism as a separate classification. This would not only streamline decision-making, but also align research with current clinical trends, allowing for a clearer assessment of PGT-A’s impact on reproductive outcomes. The clinical relevance of high-mosaic embryos—particularly in cases where no euploid embryos are available—remains an important area for investigation. Future trials should also consider the evolving approach of focusing solely on meiotic or uniformly aneuploid embryos, eliminating the need to account for mosaicism. By excluding mosaicism from the selection criteria, future studies can reduce uncertainty in clinical decision-making, enhance the reliability of PGT-A results, and better evaluate the true impact of embryo selection on implantation and live birth rates. This approach may lead to more standardized and reproducible outcomes in IVF research.

### 4.4. Generalizability

Advanced reproductive age is a well-established factor affecting reproductive outcomes, primarily due to declining oocyte quality and quantity. In our study, patients who did not proceed to randomization were generally older and had a lower embryo yield, highlighting a crucial yet often overlooked subgroup. These women typically present with more complex clinical profiles—such as diminished ovarian reserve and poorer embryo quality—which place them at higher risk for suboptimal outcomes. Paradoxically, women with poorer prognosis may benefit most from PGT-A, yet are often excluded from RCTs, creating a critical evidence gap. Their low oocyte yield and limited embryos for biopsy highlight the challenge of meeting strict eligibility criteria, such as requiring three high-quality embryos. To improve generalizability and clinical relevance, future RCTs should include these patients and explore tailored interventions that address their unique reproductive challenges. Excluding them risks incorrectly estimating PGT-A’s efficacy. Lowering the embryo threshold from three to two could expand eligibility—particularly for older women—enhancing trial power and providing more representative data on PGT-A’s real-world effectiveness.

The goal of PGT-A is to support the birth of a healthy child by reducing implantation failures and miscarriages. In our trial, 82% of women in the PGT-A group achieved clinical pregnancy after the first embryo transfer, compared to 70% in the control group. Although this showed a favorable trend, the difference was not statistically significant. While our pilot study focused on a clinical pregnancy, live birth remains the most meaningful patient-centred outcome and should be a primary endpoint in future RCTs [38].

### 4.5. Future Directions

Our pilot RCT had several strengths. It was the first to focus exclusively on women over 35—a group underrepresented in previous PGT-A trials—allowing us to assess the feasibility of studying this population. We also used an intention-to-treat (ITT) analysis, which enhanced the validity of our findings by minimizing bias from participant dropout or non-compliance. By adopting an ITT analysis, our analysis ensured that study findings reflect real-world effectiveness rather than idealized efficacy. Unlike per-protocol analysis, which only includes participants who strictly adhere to the study protocol, ITT preserves randomization and maintains the balance of confounding variables across study arms. This approach minimizes bias, particularly selection bias, which can arise when non-adherent participants are excluded, leading to an overestimation of treatment effects.

Furthermore, ITT reflects the actual impact of an intervention in a clinical setting, where non-adherence is common. This makes ITT analysis more generalizable and clinically relevant, as it accounts for patient behavior, treatment discontinuation, and protocol deviations—factors that inevitably influence real-world outcomes. In contrast, PP analysis risks inflating the perceived benefit of an intervention by selectively analysing only those who completed treatment, disregarding the complexities of patient adherence. This approach ensured inclusion of all randomized participants, offering a more accurate assessment of PGT-A’s effectiveness in the target population. With a 55.8% recruitment rate, the study engaged a substantial and diverse sample, enhancing external validity and generalizability to similar patient groups.

However, this study had limitations. It was non-blinded and conducted at a single centre. Future multicentre trials should standardize recruitment, randomization, interventions, and follow-up protocols to ensure consistency.

Based on our pilot trial, we recommend that future RCTs evaluating PGT-A in advanced reproductive age calculate separate sample sizes for two primary endpoints: live birth after first embryo transfer, and miscarriage rate. A superiority trial design is appropriate for both outcomes. Using pilot estimates, and assuming a two-sided significance level of 0.05 with 80–90% power, the sample sizes should be sufficient to detect a clinically meaningful improvement in live birth rates with PGT-A compared to conventional IVF. Detailed calculations are presented in Table 3. These tailored recommendations will help ensure future studies are adequately powered to assess PGT-A’s impact and support findings that are both statistically robust and clinically relevant in routine IVF practice.

## 5. Conclusions

In conclusion, it is feasible to conduct an RCT to evaluate the benefit of PGT-A in an IVF/ICSI cycle in women aged 35–42 years old. However, specific focus needs to be placed on including patients over the age of 39, and lowering the threshold for high-quality embryos from three to two will increase eligibility and participation rates among this population. In our pilot study, owing to the small sample size, we could not draw conclusions regarding the effectiveness of the two intervention methods.

## Figures and Tables

**Table 1:** Patient characteristics by treatment assignment.

*n* (Column %) or Median (Q_1_, Q_3_)	Total	Standard of Care	PGT-A	*p*-Value
	*n* = 100	*n* = 50	*n* = 50	
Female age	37.0 (35.0, 39.0)	37.0 (35.0, 39.0)	36.0 (35.0, 38.0)	0.074
Female ethnicity				0.34
Caucasian	83 (83.0)	45 (90.0)	38 (76.0)	
Asian	13 (13.0)	4 (8.0)	9 (18.0)	
Black	2 (2.0)	1 (2.0)	1 (2.0)	
Arab	1 (1.0)	0 (0.0)	1 (2.0)	
Other/Unknown	1 (1.0)	0 (0.0)	1 (2.0)	
Male age	39.0 (36.0, 41.0)	39.0 (36.0, 41.0)	39.0 (37.0, 41.0)	0.88
Male ethnicity				0.72
Caucasian	85 (85.0)	43 (86.0)	42 (84.0)	
Asian	9 (9.0)	4 (8.0)	5 (10.0)	
Black	5 (5.0)	3 (6.0)	2 (4.0)	
Arab	1 (1.0)	0 (0.0)	1 (2.0)	
AMH	17.6 (10.2, 30.3)	15.2 (8.6, 25.5)	20.9 (10.8, 35.8)	0.18
BMI	23.8 (21.5, 27.2)	23.8 (21.8, 27.2)	23.8 (21.2, 27.3)	0.78
Nulliparous	86 (86.0)	44 (88.0)	42 (84.0)	0.56
Cause of infertility				0.61
Unexplained	46 (46.0)	23 (46.0)	23 (46.0)	
Male factor	17 (17.0)	9 (18.0)	8 (16.0)	
Low ovarian reserve	13 (13.0)	9 (18.0)	4 (8.0)	
Anovulation	14 (14.0)	5 (10.0)	9 (18.0)	
Combination of male and female factors	7 (7.0)	3 (6.0)	4 (8.0)	
Tubal	3 (3.0)	1 (2.0)	2 (4.0)	
Insemination				0.29
ICSI	38 (38.0)	21 (42.0)	17 (34.0)	
IVF	60 (60.0)	29 (58.0)	31 (62.0)	
Mixed	2 (2.0)	0 (0.0)	2 (4.0)	
Dose of FSH	300.0 (225.0, 375.0)	300.0 (225.0, 375.0)	225.0 (225.0, 300.0)	0.16
Number of days of stimulation	10.0 (10.0, 12.0)	11.0 (9.0, 12.0)	10.0 (10.0, 12.0)	0.24
Number of eggs collected	17.0 (12.0, 20.5)	17.0 (12.0, 21.0)	17.0 (12.0, 20.0)	0.94
Number of matured oocytes	10.0 (7.0, 13.5)	10.5 (7.0, 13.0)	10.0 (7.0, 14.0)	0.86
Number of embryos biopsied/available	5.0 (3.0, 8.0)	4.5 (3.0, 8.0)	5.0 (3.0, 8.0)	0.85

**Table 2:** Outcomes by treatment assignment.

*n* (Column %)	Total	Standard of Care	PGT-A	*p*-Value
	*n* = 100	*n* = 50	*n* = 50	
Outcome of first embryo transfer				0.39
Clinical pregnancy	45 (45.0)	20 (40.0)	25 (50.0)	
Not pregnant	21 (21.0)	15 (30.0)	8 (16.0)	
Pregnancy loss				
Biochemical pregnancy loss	22 (22.0)	11 (22.0)	11 (22.0)	
Clinical Miscarriage	10 (10.0)	4 (8.0)	6 (12.0)	
Live birth on first transfer	44 (44.0)	19 (38.0)	25 (50.0)	0.23
Live birth within 3 transfers	62 (62.0)	26 (52.0)	36 (72.0)	0.039

**Table 3:** Odds ratios for clinical outcomes on first transfer of PGT-A embryos (vs. standard of care) by analytic approach and sample size estimates for future trials.

Outcome	PGT-A Group (*n* = 50)	Non-PGT-A Group (*n* = 50)	Intention-to-Treat Analysis (*n* = 100)		Per Protocol Analysis (*n* = 94)		Proposed Sample Size for Future Trials
			Unadjusted	Adjusted	Unadjusted	Unadjusted	
Ongoing clinical pregnancy after first embryo transfer	25	20	OR 1.39 95% CI: 0.64–3.03 *p*-value = 0.44	OR 1.31 95% CI: 0.59–2.29 *p*-value = 0.52	OR 1.46 95% CI: 0.65–3.28 *p*-value = 0.41	OR 1.38 95% CI: 0.59–3.24 *p*-value = 0.46	386
Clinical miscarriage after first embryo transfer	6	4	OR 1.57 95% CI: 0.41–5.94 *p*-value = 0.51	OR 1.20 95% CI: 0.52–2.79 *p*-value = 0.83	OR 1.61 95% CI: 0.42–6.12 *p*-value = 0.52	OR 1.92 95% CI: 0.47–7.79 *p*-value = 0.36	2000
Live birth after first embryo transfer	25	19	OR 1.63 95% CI: 0.74–3.62 *p*-value = 0.23	OR 1.47 95% CI: 0.61–3.54 *p*-value = 0.39	OR 1.59 95% CI: 0.71–3.59 *p*-value = 0.26	OR 1.49 95% CI: 0.61–3.66 *p*-value = 0.38	408
Live birth after a maximum of 3 embryo transfers	36	26	OR 2.37 95% CI: 1.04–5.44 *p*-value = 0.04	OR 2.26 95% CI: 0.91–5.60 *p*-value = 0.08	OR 2.47 95% CI: 1.04–5.87 *p*-value = 0.04	OR 2.50 95% CI: 0.96–6.51 *p*-value = 0.06	Not applicable

## Data Availability

The original contributions presented in this study are included in the article/Supplementary Material. Further inquiries can be directed to the corresponding author.

## Supplementary Materials

The following supporting information can be downloaded at: https://www.mdpi.com/article/10.3390/jcm14145166/s1, Table S1: Characteristic of patients eligible for randomization vs not eligible for randomization; Table S2: Analysis of clinical outcomes of patients eligible for randomization vs not eligible for randomization.
