# Supplementary material for: Preimplantation Genetic Testing for Aneuploidy Versus Morphological Selection in Women Aged 35–42: Results of a Pilot Randomized Controlled Trial
Source: J Clin Med. 2025 Jul 21;14(14):5166. doi: 10.3390/jcm14145166 (PMC12295310; doi:10.3390/jcm14145166)
Supplement: Supplementary file 1 [file jcm-14-05166-s001.zip › jcm-3731167-supplementary.pdf]

Table S1: Characteristic of patients eligible for randomization vs not eligible for randomization

**n (Column %) or Median (Q_1,Q_3)**	**Not Randomized (N=38)**	**Randomized (N=100)**
Female age	39.0 (38.0, 40.0)	37.0 (35.0, 39.0)
Female ethnicity		
Caucasian	30 (85.7)	83 (83.8)
Asian	3 (8.6)	12 (12.1)
Black	2 (5.7)	2 (2.0)
Arab	0 (0.0)	1 (1.0)
Other/Unknown	0 (0.0)	1 (1.0)
Male age	40.0 (37.0, 42.0)	39.0 (36.0, 41.0)
Male ethnicity		
Caucasian	28 (80.0)	84 (84.8)
Asian	3 (8.6)	9 (9.1)
Black	1 (2.9)	5 (5.1)
Arab	0 (0.0)	1 (1.0)
Other/Unknown	3 (8.6)	0 (0.0)
AMH	8.2 (5.3, 14.2)	17.2 (10.2, 28.9)
BMI	24.2 (22.1, 26.4)	23.9 (21.7, 27.2)
Nulliparous	35 (100.0)	85 (85.9)
Cause of infertility		
Unexplained	8 (22.9)	46 (46.5)
Male factor	14 (40.0)	17 (17.2)
Low ovarian reserve	8 (22.9)	13 (13.1)
Anovulation	0 (0.0)	13 (13.1)
Combination of male and female factors	0 (0.0)	7 (7.1)
Tubal	2 (5.7)	3 (3.0)
Endometriosis	2 (5.7)	0 (0.0)
Recurrent early pregnancy loss	1 (2.9)	0 (0.0)
Insemination		
ICSI	15 (42.9)7	38 (38.4)
IVF	19 (54.3)	59 (59.6)
Unknown	1 (2.9)	2 (2.0)
Daily Dose of FSH	375.0 (300.0, 450.0)	300.0 (225.0, 375.0)
Number of days of stimulation	10.0 (9.0, 11.0)	10.0 (10.0, 12.0)
Number of eggs collected	7.0 (5.0, 11.0)	17.0 (12.0, 20.0)
Number of matured oocytes	3.0 (2.0, 6.0)	10.0 (7.0, 14.0)
Number of embryos biopsied/available	1.0 (1.0, 2.0)	5.0 (3.0, 8.0)

Table S2: Analysis of clinical outcomes of patients eligible for randomization vs not eligible for randomization

Outcome	Non Randomized (n=38)	Randomized (n=100)	OR (95% CI)
Clinical pregnancy rate	6	45	OR 6.97<br>95% CI: 3.04-16.01<br>p-value = <0.002
Clinical miscarriage	7	10	OR 2.03<br>95% CI: 0.76-5.43<br>p-value = 0.082
Live birth rate after first embryo transfer	3	44	OR 8.90<br>95% CI: 2.56-30.92<br>p-value = 0.001
Cumulative live birth rate after a maximum of 3 embryo transfers within 1 year of randomization	4	62	OR 13.46<br>95% CI: 4.42-40.99<br>p-value = <0.001
